# Supplementary figures and images for: Evasion of serum antibodies and complement by Salmonella Typhi and Paratyphi A
Source: PLoS Pathog. 2025 May 2;21(5):e1012917. doi: 10.1371/journal.ppat.1012917 (PMC12068720; doi:10.1371/journal.ppat.1012917)

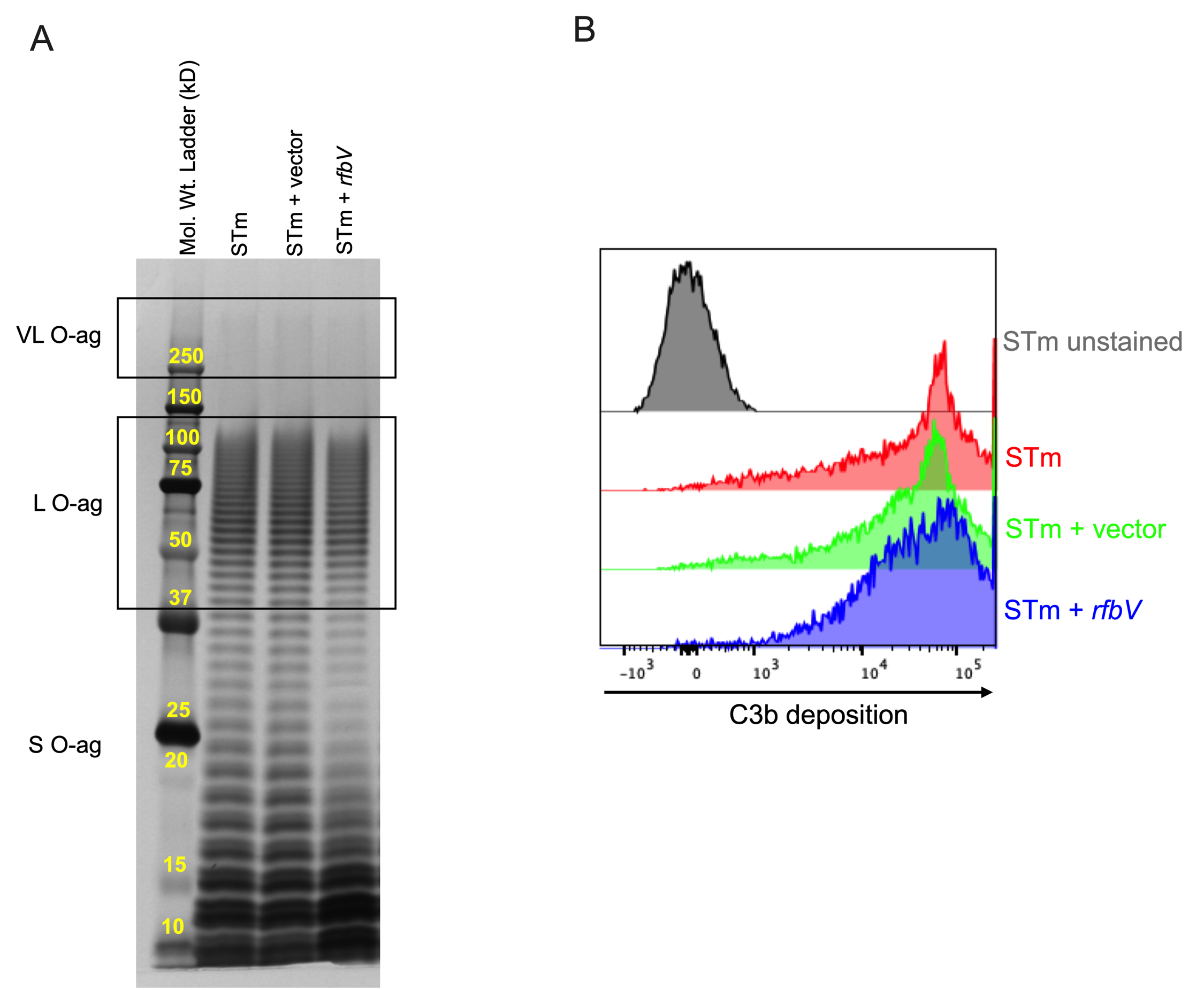

Supplement: S1 Fig — Typhimurium does not increase VL O-ag production.(A) LPS extraction followed by gel electrophoresis and silver staining shows that constitutive rfbV expression in S. Typhimurium does not increase VL O-ag production nor (B) decrease C3b deposition. STm, S. Typhimurium. (TIF) [file ppat.1012917.s001.tif]
