# Supplementary material for: Evasion of serum antibodies and complement by Salmonella Typhi and Paratyphi A
Source: PLoS Pathog. 2025 May 2;21(5):e1012917. doi: 10.1371/journal.ppat.1012917 (PMC12068720; doi:10.1371/journal.ppat.1012917)
Supplement: S1 Table — (PDF) [file ppat.1012917.s002.pdf]

Table S1. Bacterial strains and plasmids used in the study.

IDENTIFIER	BACTERIAL STRAIN / PLASMID	RELEVANT PROPERTIES	SOURCE / REFERENCE
FEG1	*Salmonella enterica* serovar Typhimurium 14028s	Wild-type with long and very long O-antigen length regulators	S. Miller
FEG11	*S. enterica* 14028s Δ*wzzB*::*tetRA*	Tetracycline resistant; long O-antigen length regulator deleted	This study
FEG9	*S. enterica* 14028s Δ*fepE*::*FRTkanFRT*	Kanamycin resistant; very long O-antigen length regulator deleted	This study
FEG12	*S. enterica* 14028s Δ*wzzB*::*tetRA* Δ*fepE*::*FRTkanFRT*	Tetracycline and kanamycin resistant; long and very long O-antigen length regulators deleted	This study
FEG21	*S. enterica* 14028s Δ*wzzB*:: $wzzB_{SPa}$	Contains *wzzB* allele from Paratyphi A	This study
FEG22	*S. enterica* 14028s Δ*wzzB*:: $wzzB_{SPa}$ Δ*fepE*::*FRTkanFRT*	Kanamycin resistant; contains *wzzB* allele from Paratyphi A; very long O-antigen length regulator deleted	This study
TY369	*Salmonella enterica* serovar Typhi Ty2 JSG624	Wild-type with long O-antigen length regulator and Vi capsule	J. Gunn
TY437	*S. enterica* Ty2 Δ*wzzB*::*tetRA*	Tetracycline resistant; long O-antigen length regulator deleted	This study
TY76	*S. enterica* Ty2 Δ*vexA*::*FRTkanFRT*	Kanamycin resistant; Vi capsule export inhibited	[1]

Table S1. Bacterial strains and plasmids used in the study.

IDENTIFIER	BACTERIAL STRAIN / PLASMID	RELEVANT PROPERTIES	SOURCE / REFERENCE
TY438	*S. enterica* Ty2 ΔwzzB::*tetRA* Δ*vexA*::*FRTkanFRT*	Tetracycline and kanamycin resistant; long O-antigen length regulator deleted and Vi capsule export inhibited	This study
TY363	*Salmonella enterica* serovar Paratyphi A ATCC 9150	Wild-type with long and very-long O-antigen length regulators	American Type Culture Collection
TY441	*S. enterica* Paratyphi A ATCC 9150 Δ*wzzB*::*tetRA*	Tetracycline resistant; long O-antigen length regulator deleted	This study
TY379	*S. enterica* Paratyphi A ATCC 9150 Δ*fepE*::*FRTkanFRT*	Kanamycin resistant; very-long O-antigen length regulator deleted	This study
TY442	*S. enterica* Paratyphi A ATCC 9150 Δ*wzzB*::*tetRA* Δ*fepE*::*FRTkanFRT*	Tetracycline and kanamycin resistant; long and very-long O-antigen length regulators deleted	This study
TY526	*S. enterica* Paratyphi A ATCC 9150 Δ*wzzB*::$wzzB_{STm}$	Contains *wzzB* allele from Typhimurium	This study
TY552	*S. enterica* Paratyphi A ATCC 9150 Δ*rfbE*::$rfbE_{STy}$	Contains *rfbE* allele from Typhi	This study
TY553	*S. enterica* Paratyphi A ATCC 9150 Δ*wzzB*::$wzzB_{STm}$ Δ*rfbE*::$rfbE_{STy}$	Contains *wzzB* allele from Typhimurium and *rfbE* allele from Typhi	This study
TY527	*S. enterica* Paratyphi A ATCC 9150 Δ*wzzB*::$wzzB_{STm}$ Δ*fepE*::*FRTkanFRT*	Kanamycin resistant; contains *wzzB* allele from Typhimurium; very-long O-antigen length regulator deleted	This study

Table S1. Bacterial strains and plasmids used in the study.

IDENTIFIER	BACTERIAL STRAIN / PLASMID	RELEVANT PROPERTIES	SOURCE / REFERENCE
TY554	*S. enterica* Paratyphi A ATCC 9150 Δ*wzzB*::*wzzB*$_{STy}$ Δ*fepE*::*FRTkanFRT*	Kanamycin resistant; contains *wzzB* allele from Typhi; very-long O-antigen length regulator deleted	This study
TY555	*S. enterica* Paratyphi A ATCC 9150 Δ*wzzB*::*wzzB*$_{STy}$ (R98C) Δ*fepE*::*FRTkanFRT*	Kanamycin resistant; contains *wzzB* allele from Typhi with R98C amino acid change; very-long O-antigen length regulator deleted	This study
TY556	*S. enterica* Paratyphi A ATCC 9150 Δ*wzzB*::*wzzB*$_{STy}$ (E158K) Δ*fepE*::*FRTkanFRT*	Kanamycin resistant; contains *wzzB* allele from Typhi with E158K amino acid change; very-long O-antigen length regulator deleted	This study
TY557	*S. enterica* Paratyphi A ATCC 9150 Δ*wzzB*::*wzzB*$_{STy}$ (T177M) Δ*fepE*::*FRTkanFRT*	Kanamycin resistant; contains *wzzB* allele from Typhi with T177M amino acid change; very-long O-antigen length regulator deleted	This study
TY571	*S. enterica* Paratyphi A ATCC 9150 Δ*waaL*::*FRTkanFRT*	Kanamycin resistant; O-antigen ligase deleted	This study
JK75	*Escherichia coli* DH5α pBSIISK(+)	Ampicillin resistant; harbors PBSIISK(+)	J. Karlinsey
FEG33	*Escherichia coli* XL-10 Ultracompetent pBSIISK(+)_*wzzB*$_{STy}$	Ampicillin resistant; harbors plasmid containing Typhi *wzzB* constructed with Gibson assembly	This study

Table S1. Bacterial strains and plasmids used in the study.

IDENTIFIER	BACTERIAL STRAIN / PLASMID	RELEVANT PROPERTIES	SOURCE / REFERENCE
FEG36	*Escherichia coli* XL-10 Ultracompetent pBSIISK(+)_*wzzB*$_{STy}$ (R98C)	Ampicillin resistant; harbors plasmid containing Typhi *wzzB* with R98C amino acid change	This study
FEG38	*Escherichia coli* XL-10 Ultracompetent pBSIISK(+)_*wzzB*$_{STy}$ (E158K)	Ampicillin resistant; harbors plasmid containing Typhi *wzzB* with E158K amino acid change	This study
FEG40	*Escherichia coli* XL-10 Ultracompetent pBSIISK(+)_*wzzB*$_{STy}$ (T177M)	Ampicillin resistant; harbors plasmid containing Typhi *wzzB* with T177M amino acid change	This study
JK18	*S. enterica* serovar Typhimurium LT2	Carries transposon insertion *Tn10dTc*[del-25] to generate *tetRA* amplicons	J. Roth
FEG26	*Escherichia coli* JKE201	1,6-diaminopimelic acid auxotroph used as conjugation donor of constructed pFOK plasmids	[2,3]
FEG140	*S. enterica* 14028s Δ*oafA*::*tetRA*	OafA abequose acetyltransferase deletion	This study
FEG151	*S. enterica* 14028s Δ*STM14_0650*::*FRT-*	F3gtrC glycosyltransferase deletion	This study
FEG153	*S. enterica* 14028s Δ*STM14_5054*::*FRT-*	F4gtrC glycosyltransferase deletion	This study
TY628	*S. enterica* Paratyphi A ATCC 9150 Δ*SPA0467*::*tetRA*	OafB rhamnose acetyltransferase deletion	This study
TY630	*S. enterica* Paratyphi A ATCC 9150 Δ*SPA2169*::*tetRA*	F3gtrC glycosyltransferase deletion	This study

Table S1. Bacterial strains and plasmids used in the study.

IDENTIFIER	BACTERIAL STRAIN / PLASMID	RELEVANT PROPERTIES	SOURCE / REFERENCE
TY632	*S. enterica* Paratyphi A ATCC 9150 Δ*SPA2387*::*tetRA*	F1gtrC glycosyltransferase deletion	This study
TY759	*S. enterica* Paratyphi A ATCC 9150 with single gene copy of *rfbX, rfbV,* and *rfbU*		This study
TY761	Strain TY759 with plasmid pJK770	Ampicillin resistance; low copy plasmid	This study
TY763	Strain TY759 constitutively expressing $rfb{*}U_3{*}X_3V_3$ on pJK770	Ampicillin resistance; low copy plasmid	This study
TY765	Strain TY759 constitutively expressing $rfb{*}U_2{*}X_2V_1$ on pJK770	Ampicillin resistance; low copy plasmid	This study
TY767	Strain TY759 constitutively expressing $rfb{*}U_2{*}X_2$ on pJK770	Ampicillin resistance; low copy plasmid	This study
TY769	Strain TY759 constitutively expressing $rfb{*}U_3{*}X_3$ on pJK770	Ampicillin resistance; low copy plasmid	This study
TY771	Strain TY759 constitutively expressing $rfbV_1$ on pJK770	Ampicillin resistance; low copy plasmid	This study
pKD46	Plasmid	*araC*-P_{araB}-γβ*exo* oriR101 repA101(30°C temperature sensitive) *bla*	[4]
pKD4	Plasmid	*FRTKanFRT* AmpR *oriR6Kγ*	[4]
pSIJ8	Plasmid	*pkd46, rhaRS*-P_{rha}-*FLPrecombinase bla*	[5]
pFOK	Plasmid	*oriT traJ aphA* P_{tetA}-*I-secI-sacB oriR6Kγ*	[2]
pJK770	Plasmid	pRB3-273C P_{Trc99a} $\Delta lacI_q$ *bla par* RK2	[6]

Abbreviations

STm, *Salmonella enterica* serovar Typhimurium
STy, *Salmonella enterica* serovar Typhi
SPa, *Salmonella enterica* serovar Paratyphi A

References

1. Karlinsey JE, Stepien TA, Mayho M, Singletary LA, Bingham-Ramos LK, Brehm MA, et al. Genome-wide Analysis of Salmonella enterica serovar Typhi in Humanized Mice Reveals Key Virulence Features. Cell Host Microbe. 2019;26: 426-434.e6. doi:10.1016/j.chom.2019.08.001
2. Cianfanelli FR, Cunrath O, Bumann D. Efficient dual-negative selection for bacterial genome editing. BMC Microbiol. 2020;20: 129. doi:10.1186/s12866-020-01819-2
3. Harms A, Liesch M, Körner J, Québatte M, Engel P, Dehio C. A bacterial toxin-antitoxin module is the origin of inter-bacterial and inter-kingdom effectors of Bartonella. PLoS Genet. 2017;13. doi:10.1371/journal.pgen.1007077
4. Datsenko KA, Wanner BL. One-step inactivation of chromosomal genes in Escherichia coli K-12 using PCR products. Proc Natl Acad Sci U S A. 2000;97: 6640–6645. doi:10.1073/pnas.120163297
5. Jensen SI, Lennen RM, Herrgård MJ, Nielsen AT. Seven gene deletions in seven days: Fast generation of Escherichia coli strains tolerant to acetate and osmotic stress. Sci Rep. 2015;5: 17874. doi:10.1038/srep17874
6. Stepien TA, Singletary LA, Guerra FE, Karlinsey JE, Libby SJ, Jaslow SL, et al. Nuclear factor kappa B-dependent persistence of Salmonella Typhi and Paratyphi in human macrophages. mBio. 2024;15: e0045424. doi:10.1128/mbio.00454-24
