# Supplementary material for: Evasion of serum antibodies and complement by Salmonella Typhi and Paratyphi A
Source: PLoS Pathog. 2025 May 2;21(5):e1012917. doi: 10.1371/journal.ppat.1012917 (PMC12068720; doi:10.1371/journal.ppat.1012917)
Supplement: S2 Table — (PDF) [file ppat.1012917.s003.pdf]

TABLE S2. PRIMERS USED IN THE STUDY.

PRIMER	SEQUENCE 5'-3'	PURPOSE
FEGP5	GATAGTAATACGTCTTCCGGGCGTGGGAACGAT CCGGAACTTAAGACCCACTTTCACATT	Deletion of *wzzB* in STm
FEGP6	CTTACAAGGCTTTTGGCTTATAGCTACGTAGCGC ATTGCGCTAAGCACTTGTCTCCTG	Deletion of wzzB in STm, STy, and SPa
FEGP9	GATAGTAATGCGTCTTCCGGGCGTGGGAACGAT CCGGAACTTAAGACCCACTTTCACATT	Deletion of *wzzB* in STy
FEGP10	GATAGTAATGCATCTTCCGGGCGTGGGAACGAT CCGGAACTTAAGACCCACTTTCACATT	Deletion of *wzzB* in SPa
TSP459	ATGCCATCTCTTAATGTAAAACAAGAAAAAAATC AGTCATGTGTGGCTGGAGCTGCTTC	Deletion of *fepE* in STm
TSP460	TCAGACTAACCGTTCATCTATCGCCAGCGCGTTT TCCATTCATATGAATATCCTCCTTAG	Deletion of *fepE* in STm
TYP45	ATCATCATATTACTAACGACATTTTTCCTGCTTTC GGGATGTGTAGGCTGGAGCTGCTTC	Deletion of *vexA* in STy
TYP46	TTAGTGCCGCGGGTCAAAAAGCTATCGAATGCG GCTTTCACATATGAATATCCTCCTTAG	Deletion of *vexA* in STy
JKP987	TCAGTCATTTGCAGGTTACTCACTGCCGCCCGC CAACAGTGTGTAGGCTGGAGCTGCTTC	Deletion of *fepE* in SPa
JKP988	TTACGCGAGACCATCGCGTGACGCAGTAATACG CCGCCGCCATATGAATATCCTCCTTAG	Deletion of *fepE* in SPa
FEGP70	CTGTCTCATCCCAAACCTATTGTGGAGAAAAGAT GCTAACGTGTAGGCTGGAGCTGCTTC	Deletion of *waaL* in SPa
FEGP71	ACGCGCTGATACCGTAATAAGTATCAGCGCGTT TTTTATCCATATGAATATCCTCCTTAG	Deletion of *waaL* in SPa
FEGP156	AATTTCGTCTTGTGTGGCACCTTGGAATTATAGG TAAAAATTAAGACCCACTTTCACATT	Deletion of *oafA* in STm
FEGP157	TGTAGTTTTATAAAATAAAAAGAGGGGCAAGCCC CTCTGTCTAAGCACTTGTCTCCTG	Deletion of *oafA* in STm
FEGP276	AGATTAAAAATGGTAATCGCTATCCTTATAGTTTT CTCGTGTGTAGGCTGGAGCTGCTTC	Deletion of *STM14_0650* in STm
FEGP277	TTACCCATTGGTGGCGGGGAACATTAATTATACA TGAATGCATATGAATATCCTCCTTAG	Deletion of *STM14_0650* in STm
FEGP280	GGCGATGATACTACTGGCTTTTTTGGCCATTTTT CTTATTGTGTAGGCTGGAGCTGCTTC	Deletion of *STM14_5054* in STm
FEGP281	CACATTTTGTTAAATGGCGAAGATCGCGTTTTAC GTAAAGCATATGAATATCCTCCTTAG	Deletion of *STM14_5054* in STm
FEGP159	ACCATTTTATTAATCTTTTTTTAAATTGAGGTAATT TAAGTTAAGACCCACTTTCACATT	Deletion of *oafB* in SPa

TABLE S2. PRIMERS USED IN THE STUDY.

PRIMER	SEQUENCE 5'-3'	PURPOSE
FEGP160	ATGTTCCATATTGGATTTATATGTAATAGTAACAG CCTATCTAAGCACTTGTCTCCTG	Deletion of *oafB* in SPa
FEGP162	ATGGGAAGAAAAATGGTTAACAATAGATTAAAAA TGGTAATTAAGACCCACTTTCACATT	Deletion of *SPA2169* in SPa
FEGP163	TTACCCATTGGTGGCGGGGAACATTAATTATACA TGAATGCTAAGCACTTGTCTCCTG	Deletion of *SPA2169* in SPa
FEGP165	GTTTTAAAAGTGAAATTTAATAGTAATGACAGGA TATTTATTAAGACCCACTTTCACATT	Deletion of *SPA2387* in SPa
FEGP166	TGTAAACACCCATTTTTATTTTATGGTAAATGTTC TATAGCTAAGCACTTGTCTCCTG	Deletion of *SPA2387* in SPa
FEGP11	CACTGTCTCCAGCTTCATCCTTTTTTTAGTTAGG GTATCT	Amplification of *wzzB* from STm and SPa for allelic exchange
FEGP12	GGCAAAGAAGCTTACAAGGCTTTTGGCTTATAG CTACGTA	Amplification of *wzzB* from STm and SPa for allelic exchange
FEGP60	TATCGATAAGCTTGATATCGATGACAGTGGATAG TAATGC	Amplification of *wzzB* from STy for HiFi assembly with EcoRI and BamHI digested pBSIISK(+)
FEGP61	CGGCCGCTCTAGAACTAGTGTTACAAGGCTTTT GGCTTATAG	Amplification of *wzzB* from STy for HiFi assembly with EcoRI and BamHI digested pBSIISK(+)
FEGP62	GAAAACGCAGAACTAAAGCAGCTTATAAAATTCG CCTGC	Introduction of c292t SNP in *wzzB* from STy resulting in R98C amino acid change; QuikChange Lightning Kit

TABLE S2. PRIMERS USED IN THE STUDY.

PRIMER	SEQUENCE 5'-3'	PURPOSE
FEGP63	GCAGGCGAATTTTATAAGCTGCTTTAGTTCTGCG TTTTC	Introduction of c292t SNP in *wzzB* from STy resulting in R98C amino acid change; used with QuikChange Lightning Kit
FEGP64	TCCTTAGCGACCTTTTCATCCACCTGTTGGATAT ATTCC	Introduction of g472a SNP in *wzzB* from STy resulting in E158K amino acid change; used with QuikChange Lightning Kit
FEGP65	GGAATATATCCAACAGGTGGATGAAAAGGTCGC TAAGGA	Introduction of g472a SNP in *wzzB* from STy resulting in E158K amino acid change; used with QuikChange Lightning Kit
FEGP66	GGACTCCTGCAACATTTTGGTTTGCAGCGTGAT G	Introduction of c530t SNP in *wzzB* from STy resulting in T177M amino acid change; used with QuikChange Lightning Kit
FEGP67	CATCACGCTGCAAACCAAAATGTTGCAGGAGTC C	Introduction of c530t SNP in *wzzB* from STy resulting in T177M amino acid change; used with QuikChange Lightning Kit

TABLE S2. PRIMERS USED IN THE STUDY.

PRIMER	SEQUENCE 5'-3'	PURPOSE
FEGP68	TTTATGACTACACTGTCTCCAGCTTCATCCTTTTTTTAGTTAGGGTATCTATGACAGTGGATAGTAATGC	Amplification of *wzzB* from constructed pBSIISK_*wzzB*$_{STy}$ for allelic exchange into SPa
FEGP69	TTAATGAGAAATTTTACCTTTCGAAGCCGACCACCATCCGGCAAAGAAGCTTACAAGGCTTTTGGCTTATAGC	Amplification of *wzzB* from constructed pBSIISK_*wzzB*$_{STy}$ for allelic exchange into SPa
FEGP107	AAGAATGAAATATTTTTTATAATTAAAAGATGAAGCTGACTTAAGACCCACTTTCACATT	Used with FEGP108 to amplify *tetRA* from STm Tn10dTc and replace *rfbX* to *rfbU* genes in SPA; contains 40 bp homology arms directly upstream of SPa *rfbX*
FEGP108	ACCCTGCATTATATGTGGGAATAATTAATGTTATTTTCATCTAAGCACTTGTCTCCTG	Used with FEGP107 to amplify *tetRA* from STm Tn10dTc and replace *rfbX* to *rfbU* genes in SPa; contains 40 bp homology arms directly downstream of SPa *rfbU*

TABLE S2. PRIMERS USED IN THE STUDY.

PRIMER	SEQUENCE 5'-3'	PURPOSE
FEGP129	TATCGATAAGCTTGATATCGATGTTACAAGATTAATAACTAAGTATATG	Used with FEGP130 to amplify 900 bp homology region upstream of SPa *rfbX;* primer overlaps with BamHI and EcoRI restriction enzyme digested pFOK for HiFi assembly into pFOK plasmid
FEGP130	GTTTCCTCACGTCAGCTTCATCTTTTAATTATAAAAAATATTTC	Used with FEGP129 to amplify 900 bp homology region upstream of SPa *rfbX* for HiFi assembly into pFOK plasmid
FEGP131	TGAAGCTGACGTGAGGAAACTGAGGTTG	Used with FEGP132 to amplify *rfbX* through *rfbY* from STy for HiFi assembly; primer overlaps with primer FEGP130 for HiFi assembly
FEGP132	TGTTATTTTCATTTATTTTGATTCCGCCAAC	Used with FEGP131 to amplify *rfbX* through *rfbY* from STy for HiFi assembly; primer overlaps with primer FEGP133 for HiFi assembly

TABLE S2. PRIMERS USED IN THE STUDY.

PRIMER	SEQUENCE 5'-3'	PURPOSE
FEGP133	ATCAAAATAAATGAAAATAACATTAATTATTCCCAC	Used with FEGP134 to amplify 900bp homology region downstream of SPa *rfbU*; primer overlaps with primer FEGP132 for HiFi assembly
FEGP134	CGGCCGCTCTAGAACTAGTGACTCTTGTACATGCTAAAATAG	Used with FEGP133 to amplify 900bp homology region downstream of SPa *rfbU*; primer overlaps with BamHI and EcoRI restriction enzyme digested pFOK for HiFi assembly into pFOK plasmid
FEGP252	ATTTCACACAGGAAACAGACCATGCTTATATCATTTTGTATTCCAAC	Used with primer FEGP253 to amplify *rfbV* from STm and ligate to NcoI restriction enzyme digested pJK770 by HiFi assembly
FEGP253	CGGGTACCGAGCTCGAATTCCTACTTAATTATCCGTTTAGTAAAATTCTTAAG	Used with primer FEGP252 to amplify *rfbV* from STm and ligate to NcoI restriction enzyme digested pJK770 by HiFi assembly

TABLE S2. PRIMERS USED IN THE STUDY.

PRIMER	SEQUENCE 5'-3'	PURPOSE
FEGP254	ATTTCACACAGGAAACAGACCATGAAGGTATCATTTTGTATCCC	Used with primer FEGP255 to amplify *rfbV* from SPa and ligate to NcoI restriction enzyme digested pJK770 by HiFi assembly
FEGP255	CGGGTACCGAGCTCGAATTCCTATGAAAATATTTTTTTTATTACCATTTTTGC	Used with primer FEGP254 to amplify *rfbV* from SPa and ligate to NcoI restriction enzyme digested pJK770 by HiFi assembly
FEGP256	ATTTCACACAGGAAACAGACCATGATCGTTAACCTATCACG	Used with primer FEGP257 to amplify *rfb*U*XV* from SPa and ligate to NcoI restriction enzyme digested pJK770 by HiFi assembly
FEGP257	CGGGTACCGAGCTCGAATTCCTATGAAAATATTTTTTTTATTACCATTTTTGC	Used with primer FEGP256 to amplify *rfb*U*XV* from SPa and ligate to NcoI restriction enzyme digested pJK770 by HiFi assembly
FEGP258	ATTTCACACAGGAAACAGACCATGATCGTTAACCTATCACG	Used with primer FEGP259 to amplify *rfb*U*X* from SPa and ligate to NcoI restriction enzyme digested pJK770 by HiFi assembly

TABLE S2. PRIMERS USED IN THE STUDY.

PRIMER	SEQUENCE 5'-3'	PURPOSE
FEGP259	CGGGTACCGAGCTCGAATTCTTATGCTAGCCTTTTACTCTTATAC	Used with primer FEGP258 to amplify *rfb*U*X* from SPa and ligate to NcoI restriction enzyme digested pJK770 by HiFi assembly
FEGP260	ATTTCACACAGGAAACAGACCATGCCATCTCTTAATGTAAAACAAG	Used with primer FEGP261 to amplify *fepE* from STm and ligate to NcoI restriction enzyme digested pJK770 by HiFi assembly
FEGP261	CGGGTACCGAGCTCGAATTCTCAGACTAACCGTTCATCTATC	Used with primer FEGP260 to amplify *fepE* from STm and ligate to NcoI restriction enzyme digested pJK770 by HiFi assembly

ABBREVIATIONS

STm, *Salmonella enterica* serovar Typhimurium strain 14028s
STy, *Salmonella enterica* serovar Typhi strain Ty2
SPa, *Salmonella enterica* serovar Paratyphi A strain ATCC 9150
SNP, single nucleotide polymorphism
