# Supplementary material for: Evasion of serum antibodies and complement by Salmonella Typhi and Paratyphi A
Source: PLoS Pathog. 2025 May 2;21(5):e1012917. doi: 10.1371/journal.ppat.1012917 (PMC12068720; doi:10.1371/journal.ppat.1012917)
Supplement: Attachment — Submitted filename: Response to Reviewers.docx [file ppat.1012917.s006.docx]

**Part II – Major Issues: Key Experiments Required for Acceptance**

Reviewer #1: The points below do not necessarily require new experiments but nevertheless need to be considered based on the data and the controls provided.

1) Line 207-210: “Increased IgM binding to STy occurs in the absence of Vi capsule, but not in strains lacking both the Vi capsule and long O-antigen, suggesting that IgM binds to long O-antigen in the absence of Vi capsule.” The conclusion does not appear to be fully supported by the data presented. IgM binds STy, STy wzzB, and STy wzzBvexA to similar degree and they are all higher than the unstained control (Fig 1E). This would suggest that IgM is not exclusively binding long O-antigen unless the ~250 MdFI is non-specific. To address the possibility of non-specific binding, flow cytometry needs a proper isotype control. For the PE anti-C3b/iC3b antibody, BioLegend has an isotype control (Cat # 400113). For AF488 anti-human IgM (Cat#400132). The catalog # listed for the anti-human IgG may be a typo.

Response: We agree with Reviewer #1’s interpretation of these data and the observation that IgM is not exclusively binding long O-antigen. This is supported by pilot experiments we performed with isotype controls that showed minimal binding of isotype controls compared to unstained controls (shown in figures below). Importantly, wildtype *S.* Typhi does bind more IgM compared to the isotype control, suggesting that some IgM epitopes are accessible even in the presence of the Vi capsule (figure below).

Isotype controls. **A**. Unstained *Salmonella* (black histogram) compared to isotype control stained (red histogram) and AF488 anti-IgM stained *S.* Typhi (blue histogram). **B**. Isotype control stained *S.* Typhi and *S.* Typhi ∆*vexA.*

We did not mean to imply that IgM binds exclusively to long O-antigen in *S.* Typhi. Nevertheless, increased IgM binding observed in the absence of Vi is most likely due to greater exposure of long O-antigen epitopes, because the absence of both Vi and long O-antigen restores IgM binding back to wildtype levels (figure 1E). To clarify this point, we will modify the text as follows:
“Although some IgM binding of wildtype *S.* Typhi is observed, increased IgM binding is seen in the absence of Vi capsule, but not in strains lacking both Vi capsule and L O-ag, suggesting that the increased IgM binding is attributable to unmasking of L O-ag epitopes in the absence of Vi capsule.”

The AF488 anti-human IgG antibody catalog #409322 is correct. However, BioLegend appears to have discontinued this clone and recommends clone QA19A42 (catalog #366921), which a BioLegend representative states performs in a manner that is consistently similar to the discontinued clone. We have included the alternative clone and catalog number in the Methods.

2) Line 289-290: “Previous studies have shown that the composition of O-antigen can impact C3b deposition [46-48].” Ref 46 and 48 are about the alternative pathway of complement activation. For this manuscript, mechanistically, is this classical, alternate, or lectin pathway activation of complement? If classical, is this IgM- or IgG-dependent? In Figure 4A-D, IgM binding does not strongly correlate with C3b deposition. IgG binding MdFI seems to match %C3b+ but not C3b deposition. Related, deletion of OafA in STm didn’t impact C5a release but increased IgM binding (Fig 6F, G). This suggests that Ig binding and complement binding are not completely in sync and therefore not entirely the classical pathway. Complement technology has various complement depleted-human sera such as C1q-depleted serum (Cat#A300) that could help address this. It is likely a mix of pathways for all serovars but it’s possible that one serovar may lean more towards the alternative pathway. Even if complement deposition is primarily due to alternative pathway, IgG and IgM binding still opsonize the bacteria and could be discussed.

Response: We appreciate these comments. The mechanisms of complement activation by the *S. enterica* serovars analyzed in this study is of considerable interest. We agree that this activation is likely to involve multiple pathways with different rates of reaction. We elected not to analyze the complex mechanism(s) of complement activation in detail in the current study because our primary goal was to identify and compare the bacterial surface factors responsible for enhancing or preventing complement deposition among the different serovars. The lack of a clear correlation between antibody binding and complement deposition and activation was somewhat unexpected and suggests that multiple pathways are likely to be responsible for complement activation. We also think it is possible that IgM and IgG binding do not entirely correlate with complement activation because activation by the classical pathway requires multivalent antibody binding for optimal activation. Biophysical studies beyond the scope of the present study will be required to determine how efficiently antibodies bind to O-antigen chains of different lengths to initiate complement activation. We are also pursuing experiments with defined depleted sera to analyze the role of opsonization and complement deposition and activation on interactions with host immune cells.

We have included the following comment to conclude the third paragraph of the Discussion to address these issues:
“We did not observe a clear correlation between antibody binding and complement activation, suggesting a contribution from additional mechanisms of activation (e.g., the alternative and mannose-binding lectin pathways). Biophysical studies may be required to determine how O-ag length affects antibody binding and initiation of complement activation. We hypothesize that O-ag length may affect the multivalent antibody binding required for complement activation. In addition, studies are underway to determine how complement activation and opsonization affect interaction of host cells with nontyphoidal and enteric fever *S. enterica* serovars.”

Reviewer #2: There are no major issues - in Minor Issues I have raised some methodological issues. I the key Figure 2A, it is difficult for me to see the detail.

Response: The Minor Issues are addressed below. The main point of Figure 2A is the lack of long O-Ag in *S.* Paratyphi A compared to *S.* Typhimurium and *S.* Typhi, which appears clear to us. In addition, *S.* Paratyphi A produces greater quantities of very long O-antigen than *S.* Typhimurium. We hope that this will be more easily visualized in the final higher resolution figures.

**Part III – Minor Issues: Editorial and Data Presentation Modifications**

Reviewer #1: 3) Figure 1A: If 10^-8 is the limit of detection, please mark it as such.

Response: Thank you for the suggestion. We have included the following statement in the figure legends with serum bactericidal assays: “An 8-log reduction in bacterial survival is the limit of detection for serum bactericidal assays.”

4) Fig 1A, B and Fig 7E are the only figures referenced in the discussion although data of the entire manuscript is discussed. Please update referenced figures for consistency in the text.

Response: We appreciate this suggestion and have referenced the appropriate figures in the Discussion for greater consistency.

5) It is not clear in the figure legends if “independent” is referring to independent experiments or biological replicates.

Response: The experiments are biological replicates. We have changed “independent” to “biological” replicates in the figure legends for clarity.


6) While serum bactericidal assays are commonly done with Salmonella cultured in LB, media mimicking the SCV has been shown to alter O-antigen of Salmonella Typhimurium (PMID 29866904). Could the authors add some discussion about this topic.

Response: We appreciate bringing attention to this important observation and agree that it is relevant to mention in the Discussion, particularly since a recent study (bioRxiv pre-print, PMID 39574608) suggests that the SCV increases expression of *pgtE*, encoding a protease that can cleave complement proteins, which may compensate for reductions in O-antigen length. We have included the following comment in the Discussion with references cited: “Serum bactericidal assays are commonly performed with *Salmonella* cultured in LB medium. Here, we show clear differences in the role of O-ag length in conferring serum resistance to nontyphoidal and enteric fever *S. enterica* serovars cultured in LB. Future studies should also analyze serum resistance of different serovars under conditions that mimic the *Salmonella-*containing vacuole, which has been shown to affect O-ag length and resistance to complement killing by increasing the expression of complement-cleaving proteases.”

Reviewer #2: Comments and Questions

1. Abstract – long and very long O antigen polymers need to be more accurately described and a suitable abbreviation found. The number of repetitive sugars, or apparent MW in SDS could be used to define ranges for each of the pathogens. Given the plasticity in chain length and loose control over polymerisation rate (which yields something of a normal distribution) getting the chemical differences between long and very long out of the way early, would make the paper more readable. Importantly, the research question(s) is clear.

Response: We appreciate the suggestion and agree that using a suitable abbreviation will make it easier to read the manuscript. As suggested, we will use L O-ag and VL O-ag for long O-antigen and very long O-antigen, respectively, throughout the manuscript. We have also included the number of repeating of O-ag units commonly described for *Salmonella* to describe the length of L and VL O-ag in the Abstract.

2. The introduction is overly detailed. Some of this detail can be referenced or, where relevant, placed in the Discussion. While the mechanism through which complement works is important, ultimately the study focusses on C3b deposition. A cartoon of the different O antigens for the 3 serovars under examination would be helpful, i.e. where the differentiating sugars like tyvelose sit in the LPS.

Response: We have simplified the Introduction by removing and summarizing some of the technical details about initial complement activation. We would like to retain the details downstream of C5 cleavage since it covers the mechanism of complement killing and anaphylatoxin production that are relevant to interpreting the results. We have also moved the cartoon model figure (Supplemental Figure 2) to the main manuscript as Figure 8, to aid the reader.

3. Results – In Figure 1, it would have been good to see some more conclusive link between the killing observed and complement or natural antibody or both. This could be done with low technology (heating serum) or specific complement removal and/or addition to say heated serum. This is an important Figure insofar as the rest of the paper builds from there. It would have been interesting to see how pooled immune sera reacted with the various forms of LPS and whether there was equivalent killing of the mutants. Granted, adjusting for the isotypes and specificities of the antibodies raised by paratyphoid, Typhimurium or typhoid infection would be difficult to QC, but this data may have provided some insights into how the vaccines work.

Response: We agree with these points. As Reviewer #2 noted, we originally expected to observe a closer correlation between bacterial killing and binding by complement or natural antibody. However, the actual results are revealing because they suggest that the location of complement deposition and antibody binding may have a significant role in determining bactericidal activity. Furthermore, our observations suggest that *S.* Typhimurium has additional complement resistance mechanisms not present in *S.* Typhi or *S.* Paratyphi A, which prevent bacterial killing despite antibody binding and complement activation and deposition, allowing *S.* Typhimurium to promote inflammation without compromising its survival.

We appreciate the reviewer’s suggestions and insights regarding immune sera and antibody QC, which will be useful for planning experiments to address this question. We are also interested in determining how *S. enterica* serovars and their O-antigen/Vi capsule-deficient mutant derivatives interact with and withstand immune sera and the effect of immune sera on interactions with immune cells. This will be a focus of future studies.

4. In S. Typhi, it is possible that there is a link between O side chain length and Vi encapsulation, as has been reported for pathogenic Klebsiellae (10.1128/spectrum.01517-21). This means that a mutation in O length in S. Typhi could also result in de-encapsulation. Some comment on this or measurement of the capsule in the WzzB might be important.

Response: We thank the reviewer for bringing this observation about Klebsiellae to our attention, demonstrating how long O-antigen might contribute to Vi capsule retention in *S.* Typhi. We agree that this is interesting since the Vi capsule is anchored to the lipid membrane by a diacyl N-acetylhexosamine (instead of the hexa-acylated lipid A to which O-antigen is attached) and can be shed, but factors contributing to capsular shedding in *S.* Typhi are not well defined. We believe that exploring this question will require more in-depth future experiments but have included a comment in the Discussion on this interesting observation, with reference, and how it might influence affect serum resistance in *S.* Typhi:
“The present study shows that both L O-ag and Vi capsule independently contribute to *S.* Typhi resistance to serum killing, but the importance of capsule retention in *S.* Typhi and its role in serum resistance is uncertain. In *Klebsiella pneumoniae,* O-ag appears to stabilize capsule retention to increase serum resistance. Future studies will be required to determine the role of L O-ag in capsule retention in *S.* Typhi and its contribution to serum resistance.”

5. In Figure 2, my copy of the figure made it very hard to see the VL Oag. I don’t know if this can be enhanced. It is hard to tell whether the levels are going up or down or staying the same. The abbreviations of the LPS forms used Figure 2 are suitable for use in the whole paper.

Response: We have increased the intensity of Figure 2C to improve its clarity and hope that the final high-resolution images will be easier to visualize. The VL O-ag of *S.* Typhimurium (STm) is difficult to see since it is present in lower quantities compared to *S.* Paratyphi A (SPa). The main observations from Figure 2A are the lack of L O-ag in *S.* Paratyphi A compared to *S.* Typhimurium and *S.* Typhi, and the increased production of VL O-ag in *S.* Paratyphi A compared to *S.* Typhimurium, which seem clear in our copy of the figure. We hope that our enhancement of Figure 2C also makes clearer the main observation from this figure, that the Arg-98 to Cys mutation of WzzB accounts for deficient long O-ag production in *S.* Paratyphi A.

6. The relative binding of the different antibody types is a very fraught experiment when unfractionated serum is used because there will be competition between high and low affinity antibodies of the different types. This means that a reading where IgG>IgM can be explained in many different ways – it can be because the IgG has greater affinity displacing IgM, or it is present in higher levels enabling competitive binding. The reagents used to detect Ig binding by antibody type may also have different affinities and hence sensitivities. The only fundamental way to determine binding is to use a monoclonal antibody that is directly conjugated and standardised. This is not a suggestion, but possibly is a limitation.

Response: We thank the reviewer for bringing this to our attention and agree that this is an important limitation. Based on this comment, we added a statement to the Discussion:

“A limitation of the present study is the use of unfractionated serum, which may contain a mixture of different antibody types with different affinities for epitopes on the bacterial surface, as well as the different affinities of conjugated antibodies used to detect antibody binding. Therefore, relative binding of antibodies may not be indicative of absolute antibody binding that would require the use of conjugated monoclonal antibodies and standardization.”

7. While the binding of C3b is done well, the question that might be asked is whether the C3b ‘on rate’ is equal where it binds to VL and L LPS, and/or whether the ‘off rate’ is equal for the different LPS types, i.e. whether the apparent differences in association of C3b represents altered kinetics. Is the stability of VL LPS the same as long LPS – i.e. would C3b bound to VL LPS be more readily shed during the analysis? There is equipment to measure binding parameters (e.g. BIACore) but it adds a complexity that is probably unnecessary. Could the binding kinetics explain differences between Paratyphi and Typhi (line 444) which may also have Vi capsule diffusion/penetration to contend with. Is there a model to determine whether the VL LPS extends beyond Vi capsule in S. Typhi, or is it present only below the capsule surface? It is possible to measure capsule thickness using AFM (10.1128/spectrum.01517-21). If C3b binds to VL LPS below the capsule surface then its ability to act as an opsonin will will be greatly diminished because the capsule could provide stearic hindrance.

Response: We appreciate these excellent points that will be addressed in future studies. Our observations suggests that the current model of C3 attachment to O-antigen should be re-examined. Hydroxyl groups of O-antigen have been proposed to serve as attachment sites for covalent linkage to C3b; however, we show that increased VL O-antigen production actually prevents C3b deposition (Figure 7). Whether this is because of steric hindrance by VL O-antigen to prevent C3b penetration or, alternatively, the stabilization of VL O-antigen hydroxyl groups to decrease the rate of attack on C3b-susceptible thioesters, remains to be determined. BIACore experiments may be able to address these questions, although such experiments may be technically challenging because purification of L and VL O-antigen will likely result in micelle formation, and it is uncertain whether the BIACore platform will be amenable to the measurement of ligand binding to non-proteinaceous substrates.

Previous studies have shown that VL O-antigen interferes with the ability of the Vi capsule to prevent complement deposition, which is the likely reason for pseudogenization of *fepE* in *S.* Typhi. We are not aware of attempts to quantify the thickness of Vi capsule or VL O-antigen, with the latter described as having >100 repeating O-antigen units. Computational modeling suggests that O-antigen structure is more complex than the linear models used for simplicity (PMID: 36802606). Future AFM studies may be informative.

8. Another comment that is not easily answerable. Do we know whether the VL and L LPS is equally distributed over the surface of the bacterium and does this matter? Are there likely to be ‘rafts’ of increased expression of the VL form (e.g. poles) or is the surface expression equally distributed – this is a difficult question to answer because there are ? no reagents that can stain VL and L forms of LPS.

Response: This is something we hope to address in future work, as relevant studies are limited. A recent study suggests that the footprint from outer membrane proteins creates channels in the O-antigen that permit antibody penetration (PMID 32051408). However, the computational modeling used in that study did not consider L or VL forms of O-antigen that contain 16-35 or more than 100 repeating units, respectively. Our results in Figure 7 suggest that increased production of VL O-antigen is sufficient to prevent complement deposition, whereas complement C3 deposition would have been anticipated if the outer membrane were exposed and susceptible hydroxyl or amine groups were present.

9. Discussion – this section is done well. The ‘even bigger’ perspective might be important here. Three evolutionarily linked serovars having evolved different mechanisms for dealing with the binding of non-immune antibody and complement to the bacterial surface. It might be reflected somewhere that S. Typhimurium is really only an accidental pathogen of humans – the transmission chains that are well mapped for S. Typhi and less clear for human infections of iNTS. If S. Typhimurium spends the majority of its existence outside of the human host (cf. S. Typhi), should we expect the relationships with human antibody to be the same or different. As importantly, how does S. Paratyphi that lack a key virulence determinant (Vi) produce a syndrome that looks like typhoid fever – from this perspective, using a Vi-encapsulated S. Paratyphi may have been instructive, and to test whether there WzzB-driven approaches to serum resistance are still observed in S. Paratyphi that carry Vi.

Response: We appreciate the positive comments. A similar experiment to the one suggested here with Vi capsule has previously been performed with *S.* Typhimurium to show that Vi inhibits complement deposition. We would anticipate that Vi expression in *S.* Paratyphi A will also inhibit complement deposition. The reviewer’s suggestion that *S.* Typhimurium is pro-inflammatory because it has not co-evolved with humans is an interesting hypothesis but seems inconsistent with the fact that it stimulates inflammatory responses in all mammalian hosts. Our concluding paragraph in the Discussion expresses our current view of the role of human antibodies and complement in nontyphoidal and enteric fever serovars. In short, nontyphoidal serovars like *S.* Typhimurium exploit antibody recognition and complement activation and deposition to induce an inflammatory state in which *S.* Typhimurium thrives by eliciting the recruitment of inflammatory cells and exploiting their products to outcompete the resident microbiota. In contrast, the enteric fever serovars evade the inflammatory response by limiting antibody recognition and complement activation and deposition. We posit that the initial avoidance of inflammation is required for the persistent infection of host cells by enteric fever serovars to initiate chronic infection. A concluding sentence has been added to the Abstract to emphasize this broad perspective.

Reviewer #3: 1. The finding that rfbV gene duplication contributes to serum resistance in S. Paratyphi is interesting, yet the mechanism by which it enhances the quantity of very long O-antigen production remains incompletely understood. Is it simply a gene dosage effect? Are the three copies of rfbV identical? What is role of the hypothetical rfbU-rfbX fusion ORF in O-antigen production and how does it affect serum resistance? The authors present an intriguing hypothesis that could easily be tested, or so it seems.

Response: We suggest that the *rfbV* gene duplication arose to overcome the inefficiency of the RfbV enzyme processing paratose after *S.* Paratyphi A lost the ability to produce tyvelose. As the reviewer suggests, this is most likely a gene dosage effect. Two of the three RfbV proteins encoded by Paratyphi A are identical, while the third RfbV differs by just one amino acid out of 333. Interestingly, the RfbV proteins of *S.* Typhi and *S.* Paratyphi A differ by a couple of amino acids, and we are performing experiments with *rfbV* allelic exchange in *S.* Typhi and *S.* Paratyphi A to determine whether O-antigen production is influenced by these differences. These experiments have been complicated by serovar-specific sequence variations in other O-antigen processing enzymes such as RfbX that may also affect the efficiency of O-antigen production. As mentioned in the Discussion, we believe that the RfbU-RfbX fusion protein of *S.* Paratyphi A retains enzymatic activity since its expression alone was able to restore some serum resistance and O-antigen production. We believe that its primary role is to accommodate the increase in RfbV activity in O-antigen production. We were unable to overexpress *rfbV* alone in *S.* Paratyphi A without the complementing vector acquiring mutations that are likely to have decreased *rfbV* expression, suggesting that excessive RfbV is detrimental unless accompanied by the RfbU-RfbX fusion protein.

2. More careful consideration should be given to the use of genetic complementation of select mutations or gene deletions.

Response: We appreciate the importance of genetic complementation of key mutations. Allelic exchange of *wzzB* in *S.* Typhimurium and *S.* Paratyphi A was performed to show that WzzB activity was responsible for the phenotype of mutant strains.

**Figure resubmission:**

While revising your submission, please upload your figure files to the Preflight Analysis and Conversion Engine (PACE) digital diagnostic tool, [https://pacev2.apexcovantage.com/](https://urldefense.com/v3/__https:/pacev2.apexcovantage.com/__;!!K-Hz7m0Vt54!iWQvLgqKaUmVDH4HakDtYYr2imHIJx3Cfu6t6m__4ZNU_TPrirbao7ZpN8A2_fC2lwXN7Ik51QuXLvA$). PACE helps ensure that figures meet PLOS requirements. To use PACE, you must first register as a user. Registration is free. Then, login and navigate to the UPLOAD tab, where you will find detailed instructions on how to use the tool. If you encounter any issues or have any questions when using PACE, please email PLOS at [figures@plos.org](mailto:figures@plos.org). Please note that Supporting Information files do not need this step. If there are other versions of figure files still present in your submission file inventory at resubmission, please replace them with the PACE-processed versions.

Response: All figures have been processed through PACE. We made a small change to Figure 7A by changing “Constructed *S.* Paratyphi with single *rfbV* gene” to “Constructed *S.* Paratyphi **A** with single *rfbV* gene” to reflect the specific serovar. We also edited Figure 8 (previously supplemental Figure 2 ) to include abbreviations for long (L) and very long (VL) O-ag, as requested by Reviewer #2.
